# Web-based online resources about adverse interactions or side effects associated with complementary and alternative medicine: a systematic review, summarization and quality assessment

**DOI:** 10.1186/s12911-020-01298-5

**Published:** 2020-11-09

**Authors:** Jeremy Y. Ng, Vanessa Munford, Harmy Thakar

**Affiliations:** Department of Health Research Methods, Evidence, and Impact, Faculty of Health Sciences, McMaster University, Michael G. DeGroote Centre for Learning and Discovery, Room 2112, 1280 Main Street West, Hamilton, ON L8S 4K1 Canada

**Keywords:** Adverse events, Complementary and alternative medicine, eHealth, Online resources, Herbal therapies, Herb-drug interactions, Quality assessment, Side effects

## Abstract

**Background:**

Given an increased global prevalence of complementary and alternative medicine (CAM) use, healthcare providers commonly seek CAM-related health information online. Numerous online resources containing CAM-specific information exist, many of which are readily available/accessible, containing information shareable with their patients. To the authors’ knowledge, no study has summarized nor assessed the quality of content contained within these online resources for at least a decade, specifically pertaining to information about adverse effects or interactions.

**Methods:**

This study provides summaries of web-based online resources that provide safety information on potential interactions or adverse effects of CAM. Specifically, clinicians are the intended users of these online resources containing patient information which they can then disseminate to their patients. All online resources were assessed for content quality using the validated rating tool, DISCERN.

**Results:**

Of 21 articles identified in our previously published scoping review, 23 online resources were eligible. DISCERN assessments suggests that online resources containing CAM-specific information vary in quality. Summed DISCERN scores had a mean of 56.13 (SD = 10.25) out of 75. Online resources with the highest total DISCERN scores across all questions included Micromedex (68.50), Merck Manual (67.50) and Drugs.com (66.50). Online resources with the lowest total scores included Drug Information (33.00), Caremark Drug Interactions (42.50) and HIV Drug Interactions (43.00). The DISCERN questions that received the highest mean score across all online resources referred to whether the risks were described for each treatment (4.66), whether the aims were clear (4.58), whether the source achieved those aims (4.58), and whether the website referred to areas of uncertainty (4.58). The DISCERN questions that received the lowest mean score across all online resources assessed whether there was discussion about no treatment being used (1.29) and how treatment choices would affect quality of life (2.00).

**Conclusion:**

This study provides a comprehensive list of online resources containing CAM-specific information. Informed by the appraisal of these resources, this study provides a summarized list of high quality, evidence-based, online resources about CAM and CAM-related adverse effects. This list of recommended resources can thereby serve as a useful reference for clinicians, researchers, and patients.

## Background

Online resources that provide information about adverse interactions or side effects associated with complementary and alternative medicine (CAM) are paramount for obtaining updated and complete information for both clinicians who encounter patients who inquire about CAM, as well as researchers with an interest in this area [1]. CAM is an umbrella term used to describe a wide range of therapies that include a large number of modalities and which originate from different parts of the world. A plethora of widely-used terms and definitions for CAM exists [2], however, the National Center for Complementary and Integrative Health (NCCIH) defines a non-mainstream practice used together with conventional medicine as “complementary”, and a non-mainstream practice used in place of conventional medicine as “alternative”. Though adverse events and side effects can arise from the use of a wide-range of CAM therapies, the majority of them involve natural products, such as dietary supplements and herbal therapies [3, 4]. It is estimated that 80% of the population internationally uses natural products [5], with that increasing up to 95% in developing countries [6]. While limited data exists regarding the prevalence of adverse events associated with dietary supplements, it has been found that the rate of natural product users who report adverse effects is approximately 12–14% in Canada and the United States [4, 7]. As such, clinicians and researchers have a responsibility to utilize CAM-related electronic resources that are accurate, up-to-date, and of high quality.

Since the advent of the internet, many online resources have since been created to serve this purpose, however, many have since ceased to exist or to be updated. A recent scoping review identified numerous reviews that have been published providing overviews or summaries of CAM-related healthcare or research resources. However, the most recent reviews providing CAM information in general were published approximately 10–20 years ago and are undoubtedly obsolete to at least some degree [8–15]. Additionally, with the exception of Boehm et al.’s review, the other CAM-related reviews did not employ a systematic search strategy, and therefore likely do not provide a comprehensive list of resources, arguably even at the time of publication [8–11].

Today, there exists more information on the internet about CAM than ever before; a search for “complementary and alternative medicine” on Google alone yields 119 million search results as of May 2020 [16]. This vast abundance of such online information is not regulated for quality, nor is it formally assessed in the literature using a validated tool, such as the DISCERN instrument [17]. This lack of quality regulation makes it challenging and time-consuming for even experienced clinicians and researchers to evaluate for quality when searching for information about CAM. Perhaps even more disadvantaged are patients who often do not have the expertise to appropriately evaluate online clinical content, and are likely to consult the internet for answers in the moment of experiencing a CAM-related adverse event or side effect. Over recent years, CAM use has become increasingly more popular among patients [18]. Amongst healthcare providers, a greater acceptance of CAM has ensued, coupled with the recognition that there is a need for clinicians to increase their knowledge of CAM [19, 20]. For these reasons in conjunction with a dramatic rise in interest in conducting CAM studies among researchers [2, 21], a more recent update incorporating a systematic search is warranted. Thus, the purpose of this study is to summarize and assess the content quality of web-based online resources providing information about adverse interactions or side effects associated with CAM [22] in order to provide clinicians and researchers with a comprehensive list of resources containing information on this topic.

## Methods

### Approach

A systematic search and scoping review published by Ng et al. in 2020 was undertaken to answer the following research question: “What eHealth technologies are assisting in identifying potential (1) adverse drug interactions with CAM, (2) adverse CAM-CAM interactions or (3) standalone CAM adverse events or side effects?” [22]. Based on our understanding of this existing knowledge gap, we elected to present our findings in the format of summaries outlining what each resource offers the user, along with DISCERN instrument quality assessments of each resource allowing clinicians and researchers to quickly choose a suitable resource for their needs.

### Step 1: Selecting eligible online resources

The research questions for the present study were as follows: “What web-based online resources are typically available and accessible to clinicians that contain patient information about adverse interactions or side effects associated with CAM?” and “What is the quality of patient health information provided by these aforementioned resources?”. For the purpose of this study, we considered an operational definition of CAM inclusive of the following 5 categories of therapies: whole medical systems; mind–body medicine; biologically based practices not usually used in conventional medicine; manipulative and body-based practices; and energy medicine [23]. While we acknowledge that the large majority of adverse interactions or side effects associated with CAM likely pertain to biologically-based practices not usually used in conventional medicine, such as dietary and herbal supplements, we did not limit our definition of CAM to this single category. Instead, we also considered other CAMs such as chiropractic or acupuncture, which also have standalone adverse interactions or side effects, in addition to contraindications with various conventional therapies. All eligible eHealth technologies derived from Ng et al.’s scoping review were re-assessed by JYN and VM, and only deemed eligible for the purpose of the current study if they were a web-based online resource that presented information on potential interactions, adverse effects, or safety risks of CAM. eHealth technologies were excluded if they were not web-based online resources or were online resources that met one or more of the following criteria: exclusively mobile-based online resources; online resources only providing information about the biochemical properties, molecular structure, or chemical compositions of CAMs (i.e. herbs, traditional Chinese medicines); or online resources that contained dead weblinks. It should be clarified that the present study did not seek to identify nor assess the quality of websites typically assessed by patients or members of the public, hence we did not search commonly used search engines such as Google.

Next, JYN and VM data extracted the following information from all included online resources: name of online resource; eligible article(s) from which it was referenced; online resource URL; year established; availability of the online resource; type of developer; whether the online resource contained information on only CAM or CAM and conventional therapies. Additionally, we provide a brief summary of each included online resource that we present in this article’s "Results" section.

### Step 2: Assessing online resource content quality

The content quality of all eligible online resources was assessed using the DISCERN instrument. While a variety of instruments exist to assess health information, we selected DISCERN as it is one of the most widely-accepted, reliable, and validated instruments. It contains a series of 16 questions that are designed to assess the quality of consumer health information found offline or on the Internet using a rating scale of 1–5 for each question. The instrument was developed by a team of researchers from the University of Oxford and the British Library. Designed for use by both healthcare professionals and the general public, the instrument is publicly available on the web (www.discern.org.uk) [17].

Prior to assessing all online resources, all three authors participated in a pilot assessment of a subset of online resources using the DISCERN assessments. After independently applying the DISCERN instrument, the authors met to discuss discrepancies in their scores. Disagreements were resolved by discussion, and in the case that consensus could not be reached, a majority vote was used to assign rating scores. Subsequently, VM and HT independently assessed each included online resource using DISCERN, then all three authors met to discuss scores and resolve discrepancies. We made every attempt to assess subscription-based online resources; if we did not have access to them through our university library system, we contacted the company, organization or individual that produced the online resource requesting trial access for the purpose of this study. Acknowledging that the DISCERN instrument was originally designed to assess the patient health information of a treatment choice (and not specifically the information pertaining to adverse events and side effects, though this is undoubtedly a large component of whether a treatment choice would be deemed suitable or not), we conducted a general quality assessment for each eligible online resource, where we looked at a large enough proportion of CAM-related patient information surrounding adverse events and side effects across each online resource to provide a holistic DISCERN score. Lastly, we acknowledge that while some online resources only contained CAM-specific information, others provided additional information about non-CAM (i.e. conventional medicine). To standardize our quality assessment, we only used the DISCERN instrument to assess the quality of CAM-specific information provided by all resources. Thus, we provide a quality assessment for resources that a clinician may want to consult when providing a patient with CAM-specific information. We did not make a judgement of quality associated with the non-CAM sections of any resources.

### Step 3: Analysis of DISCERN results

DISCERN ratings from VM and HT were combined by calculating the mean values of the author’s independent DISCERN scores. The sum of these combined scores were used to calculate the total DISCERN scores and standard deviations for each online resource. The average scores were also calculated for each DISCERN question across all online resources, as well as the standard deviation of the overall score assigned in question 16.

## Results

### ***Search results (Fig. ******1******)***

**Fig. 1:**
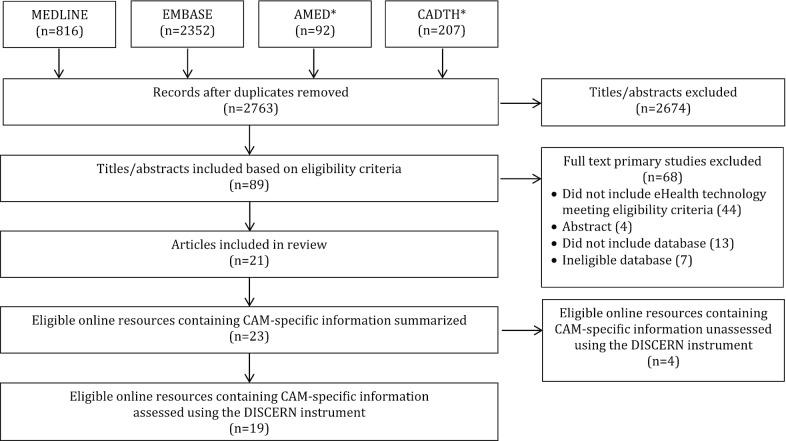
PRISMA diagram. *AMED* Allied and Complementary Medicine Database, *CADTH* Canadian Agency for Drugs and Technologies in Health, *CAM* complementary and alternative medicine

Forty-one articles from the Ng et al. scoping review were assessed for eligibility in the present study, whereby all articles referenced an electronic tool or resource containing information about CAM and CAM-related adverse effects. Of these 41 articles, 20 articles were eliminated because they developed or referenced (1) other eHealth technologies that were not web-based online resources (n = 13), or (2) online resources that were entirely mobile-based, predominantly biochemistry focused or unavailable (n = 7).Thus, a total of 21 articles were found to be eligible and included in the present study [8–14, 24–37]. A PRISMA diagram is provided in Fig. 1 of “Appendix 1”.

### Findings from eligible articles

A compiled list of the 21 eligible articles and characteristics are provided in Table 1 of “Appendix 2”. The list of eligible articles includes both primary research articles (n = 9) and review articles (n = 12). Primary research articles were designed to develop (n = 3) or evaluate (n = 6) one or more online resources containing CAM-specific information, while all secondary articles (n = 12) reviewed eHealth technologies and online resources containing CAM-specific information that met our research criteria. Articles were published from 2001 to 2019 in the United States (n = 14), UK (n = 2), China (n = 2), Singapore (n = 1), Italy (n = 1), Greece (n = 1) and Canada (n = 1).

**Table 1:** Eligible article characteristics (n = 21)

References	Article title	Study country	Study design	Article type
Allais et al. [8]	Access to databases in complementary medicine	Italy	Review of medical information resource(s)	Review
Archer et al. [24]	Development of an alert system to detect drug interactions with herbal supplements using medical record data	USA	Development of alert system prototype	Original Research
Boddy et al. [25]	Review of reliable information sources related to integrative oncology	UK	Review of medical information resource(s)	Review
Boehmer et al. [26]	Evaluating the value of a web-based natural medicine clinical decision tool at an academic medical center	USA	Evaluation of web-based clinical decision tool	Original Research
Clauson et al. [27]	Clinical decision support tools: Personal digital assistant versus online dietary supplement databases	USA	Review and evaluation of databases and personal digital assistants	Review
Faubert et al. [28]	A pilot study to compare natural health product-drug interactions in two databases in Canada	Canada	Evaluation of databases	Original Research
Fischer et al. [29]	Complementary and alternative medical reference software for personal digital assistants: Evidence of clinical applicability	USA	Evaluation of databases	Original Research
Fitzpatrick et al. [30]	Natural standard database	USA	Review of medical information resource(s)	Review
Gregory et al. [31]	Characterization of complementary and alternative medicine-related consultations in an academic drug information service	USA	Analysis of complementary and alternative medicine drug information consultations	Original Research
Jackson [9]	An overview of information resources for herbal medicinals and dietary supplements	USA	Review of medical information resource(s)	Review
Jackson et al. [10]	Resources for information on herbal medicinals and dietary supplements	USA	Review of medical information resource(s)	Review
Kiefer et al. [12]	Finding information on herbal therapy: A guide to useful sources for clinicians	USA	Review of medical information resource(s)	Review
Meyer et al. [13]	Evaluation of herbal-drug interaction data in tertiary resources	USA	Review of medical information resource(s)	Review
Molassiotis et al. [32]	Quality and safety issues of web-based information about herbal medicines in the treatment of cancer	China, UK	Review of medical information resource(s)	Review
Motl et al. [11]	Health information web sites by therapeutic category for healthcare professionals	USA	Review of medical information resource(s)	Review
Sun et al. [34]	Development of quantitative structure–activity relationship models to predict potential nephrotoxic ingredients in traditional chinese medicines	China	Development and testing of model	Original Research
Spanakis et al. [33]	PharmActa: Empowering patients to avoid clinical significant drug-herb interactions	Greece	Evaluation of mobile app	Original Research
Sweet et al. [14]	Usefulness of herbal and dietary supplement references	USA	Review of medical information resource(s)	Review
Tomasulo [35]	Natural Standard–new integrative medicine database	USA	Review of medical information resource(s)	Review
Walker et al. [36]	Evaluation of the ability of seven herbal resources to answer questions about herbal products asked in drug information centers	USA	Evaluation of databases	Original Research
Yap et al. [37]	Utilizing mobile networks for the detection of clinically relevant interactions between chemotherapy regimens and complementary and alternative medicines	Singapore	Development of an iPhone app	Original Research

### Online resource characteristics

Twenty-three web-based online resources containing CAM safety information were extracted from the eligible articles. Six of these online resources were dedicated entirely to CAM, while 17 online resources provided information on CAM as well as other conventional therapies. For many online resources, it was difficult to determine the exact date of establishment, however online resources were established as early as 1970 to as late as 2007. At least 11 online resources were established prior to 2015. All online resources were found to have been used in some form of context outside the author’s study, most often having been referenced in other papers in the literature. All online resources were available for the public to access and were either entirely free (n = 14), required a paid subscription (n = 8), or required a partial subscription (n = 1). Characteristics of all included online resources are detailed in Table 2 of “Appendix 2”.

**Table 2:** Eligible online resource characteristics (n = 23)

Name of online resource	Source eligible article(s) included in review	Online resource URL	Year established	Availability for anyone to use?	Type of developer	CAM only or CAM/conventional therapies?	How are CAM-specific adverse effects and side effects presented?
About Herbs	Boddy et al. [25]; Molassiotis et al. [32]; Yap et al. [37]	https://www.mskcc.org/cancer-care/diagnosis-treatment/symptom-management/integrative-medicine	Unclear	Yes, entirely free	Researchers	CAM only	Drug summaries (Professional and consumer versions)
American Botanical Council	Fitzpatrick [30]; Jackson et al. [10]; Kiefer et al. [12]; Motl et al. [11]	https://abc.herbalgram.org/site/PageServer	1988	Yes, partially without subscription	Council	CAM only	Monographs
Caremark Drug Interactions	Yap et al. [37]	https://cpref.goldstandard.com/inter.asp?r=8084	Unclear	Yes, entirely free	Company	Both	Drug interaction checker
Clinical Pharmacology (also known as Gold Standard)	Gregory et al. [31]	https://www.clinicalpharmacology.com/	Unclear	Yes, but only with a subscription	Company	Both	Drug interaction checker; monographs
Drug Information (formerly DrugDigest)	Motl et al. [11]	https://www.express-scripts.com/medco/consumer/ehealth/druginfo/dlmain.jsp?WC=N	Unclear	Yes, entirely free	Company	Both	Drug summaries
Drug Product Database	Motl et al. [11]	https://www.canada.ca/en/health-canada/services/drugs-health-products/drug-products/drug-product-database.html	Unclear	Yes, entirely free	Government	Both	Monographs; safety update tables
Drugs.com	Motl et al. [11]; Spanakis et al. [33]; Yap et al. [37]	https://www.drugs.com/	2001	Yes, entirely free	Company	Both	Drug interaction checker; monographs (professional and consumer versions)
Electronic Medicines Compendium	Motl et al. [11]	https://www.medicines.org.uk/emc/	1999	Yes, entirely free	Company	Both	SmPC; patient leaflets; alert cards
Epocrates (plus or pro)	Clauson et al. [27]; Fischer et al. [29]; Yap et al. [37]	https://online.epocrates.com/	Unclear	Yes, but only with a subscription	Company	Both	Drug interaction checker; monographs
Herb-Drug Interactions, NCCIH Clinical Digest	Allais et al. [8]; Boddy et al. [25]; Gregory et al. [31]; Jackson et al. [10]; Motl et al. [11]	https://nccih.nih.gov/health/providers/digest/herb-drug	2015	Yes, entirely free	Government	CAM only	Summaries provided in an herbal digest
Herbs at a Glance	Boddy et al. [25]; Jackson et al. [10]; Gregory et al. [31]; Motl et al. [11]	https://nccih.nih.gov/health/herbsataglance.htm	Unclear	Yes, entirely free	Government	CAM only	Fact sheets
HIV drug interactions	Motl et al. [11]	https://www.hiv-druginteractions.org/checker	1999	Yes, entirely free	Researchers	Both	Drug interaction checker; interaction charts
IBM Micromedex (includes DrugDex, Drug-Reax, AltMedDex)	Clauson et al. [27]; Fischer et al. [29]; Jackson [9]; Jackson et al. [10]; Kiefer [12]; Meyer et al. [13]; Walker [36]; Yap et al. [37]	https://www.micromedexsolutions.com/home/dispatch/ssl/true	Mid-1970s	Yes, but only with a subscription	Company	Both	Drug interaction checker; monographs; patient fact sheets
Lexi-Natural (includes LexiComp)	Clauson et al. [27]; Gregory et al. [31]; Fischer et al. [29]; Motl et al. [11]; Yap et al. [37]	https://webstore.lexi.com/Store/Individual-Databases/Lexi-Natural-Products	Unclear	Yes, but only with a subscription	Company	Both	Monographs; patient handouts
MedicinesComplete (includes Herbal Medicines; formerly known as the British National Formulary)	Jackson [9]; Jackson et al. [10]; Yap et al. [37]	https://about.medicinescomplete.com/publication/herbal-medicines/	Unclear	Yes, but only with a subscription	Company	Both	Monographs
Medscape	Motl et al. [11]; Spanakis et al. [33]	https://www.medscape.com/	1995	Yes, entirely free	Company	Both	Drug interaction checker; monographs
Merck Manual	Motl et al. [11]	https://www.merckmanuals.com/en-ca/	1999	Yes, entirely free	Company	Both	Drug interaction checker; monographs (professional and consumer versions)
National Cancer Institute	Boddy et al. [25]	https://www.cancer.gov/about-cancer/treatment/cam	Unclear	Yes, entirely free	Government	CAM only	Drug summaries (professional and consumer versions)
Natural Medicines (formerly Natural Medicine Comprehensive Database (NMCD) and Natural Standard Database (NSD)) (online and mobile app)	Archer et al. [24]; Boddy et al. [25]; Boehmer et al. [26]; Clauson et al. [27]; Faubert et al. [28]; Fischer et al. [29]; Fitzpatrick [30]; Gregory et al. [31]; Jackson et al. [10]; Kiefer et al. [12]; Motl et al. [11]; Sun et al. [34]; Sweet et al. [14]; Tomasulo [35]; Walker [36]; Yap et al. [37]	https://naturalmedicines.therapeuticresearch.com/	Early 2000s	Yes, but only with a subscription	Company	CAM only	Drug interaction checker; monographs; patient handouts
OncoRx Database (called Onco-Rx as a website, and OncoRx-MI as a mobile app)	Yap et al. [37]	https://www.onco-informatics.com/oncorx/	2007 (website), unclear (mobile app)	Yes, but only with a subscription	Researchers	Both	Drug interaction checker
PEPID Drug Information Database	Clauson et al. [27]; Fischer et al. [29]	https://www.pepid.com/	Unclear	Yes, but only with a subscription	Company	Both	Drug interaction checker; patient education handouts; monographs
RxList (owned by WebMD)	Motl et al. [11]; Spanakis et al. [33]	https://www.rxlist.com/script/main/hp.asp	1995	Yes, entirely free	Company	Both	Monographs
RxMed	Motl et al. [11]	https://www.rxmed.com	1994	Yes, entirely free	Company	Both	Monographs

### Summaries of included online resources

Summaries for the 23 online resources are provided below:

#### About Herbs

Website: https://www.mskcc.org/cancer-care/diagnosis-treatment/symptom-management/integrative-medicine

About Herbs is an online database managed by the Memorial Sloan Kettering Cancer Center. The database provides herb and dietary supplement monographs for patients and caregivers, as well as professional monographs for healthcare providers.

Monographs for patients/caregivers list warnings and side effects. Monographs for healthcare professionals list adverse reactions, contraindications, warnings and interactions.

#### American Botanical Council

Website: https://abc.herbalgram.org/site/PageServer

The American Botanical Council (also known as "Herbal Medicine Institute") is a non-profit organization that provides information to consumers, researchers and health professionals on herbal medicines. The website includes: ABC Clinical Guide to Herbs (online searchable database of monographs, clinical overviews and patient information sheets); HerbalGram (online journal publications); HerbClip (database of videos that summarize and critically review research, publications and marketing material of medicinal drugs); Herbal MediaWatch; (articles from various media sources regarding health and medicinal plants); product-specific and botanical ingredient monographs; HerbMed (online database of resources, articles and summaries of various herbal drugs); Adulteration Program (to provide education and prevent the ingredient and product adulteration of herbal and dietary supplements); and a medicinal plant identification database.

Monographs and drug profiles on HerbMed list adverse effects and toxicity, interactions, contraindications and relevant evidence from clinical trials about product safety.

#### Caremark Drug Interactions

Website: https://cpref.goldstandard.com/inter.asp?r=8084

Copyrighted through Gold Standard, Caremark Drug Interactions is a web-based database of drug interactions. Reports can be generated for potential interactions between prescription drugs, herbs, vitamins, over the counter drugs, caffeine, grapefruit juice, food, tobacco, alcohol and enteral feedings.

The resource contains a searchable drug interaction checker tool that provides alerts for potential drug interactions. The level of severity and possible side effects are listed for each interaction. The website indicates drugs, supplements or drug combinations that require a user to contact their healthcare provider.

#### Clinical Pharmacology (through Clinical Key)

Website: https://www.clinicalpharmacology.com/

Clinical Pharmacology is a drug information resource that is powered by Clinical Key and owned by Gold Standard. The resource contains a database of monographs for prescription drugs, over the counter products, herbal medicines and nutritional supplements. Other features include: IV compatibility tests; drug product comparisons; patient education handouts; drug product information; drug interaction reports; clinical decision support tool; drug IDentifier; resources and educational modules.

The resource contains a searchable drug interaction checker tool that generates interaction reports. Drug monographs provide information on contraindications, warnings, adverse effects and interactions with other drugs, food or supplements.

#### Drug Information (formerly DrugDigest)

Website: https://www.express-scripts.com/medco/consumer/ehealth/druginfo/dlmain.jsp?WC=N

Drug Information (formerly known as DrugDigest), is a web-based, searchable database that provides information on medications, herbal medicines and dietary supplements. The database indexes various brands, dosages and forms of a drug or supplement. Drug summaries include frequently asked questions which outline the benefits, risks and guidelines for consumers. Several drugs are indexed in the database; however, a large proportion do not have information available.

Drug summaries list potential side effects as well as other more serious adverse effects that warrant medical attention. Warnings and risks are listed which include: warnings for pregnant or breastfeeding women; interactions with other drugs or substances; instructions in cases of missed dosages; items that should be discussed with a healthcare provider before starting a drug.

#### Drug Product Database

Website: https://www.canada.ca/en/health-canada/services/drugs-health-products/drug-products/drug-product-database.html

The Drug Product Database is maintained by Health Canada and contains a searchable database of drugs authorized for sale in Canada. The database includes: availability of drugs on the market; product monographs for human drugs; labels for animal drugs; tables containing product and monograph safety updates. The database indexes drugs of various statuses including those have been approved, marketed or cancelled. A small proportion of drugs in the database have online product monographs available.

The website provides a table for each month that lists safety updates made to monographs in one or more of the following areas: contraindications; warnings and precautions; adverse reactions; drug interactions; dosage and administration; overdosage; consumer information. When a drug product monograph is available in the database, it includes information for both healthcare professionals and consumers. The content of monographs vary but commonly provide information on adverse reactions, drug interactions, contraindications, warnings and precautions.

#### Drugs.com

Website: https://www.drugs.com/

Drugs.com is a web-based resource that provides drug information to healthcare professionals and patients. The resource includes: database of consumer leaflets and professional drug monographs; drug interaction checker; FDA alerts; news centre, drug approval updates, clinical trials; pill identifier; dosage guides; drug pricing guides; symptom checker; disease reference summaries; health education videos. Drugs.com is also offered as a mobile resource, though this was not evaluated for quality in the present study.

The searchable drug interaction checker tool provides alerts for potential drug interactions. Alerts are provided for drugs, alcohol, food and disease interactions. Side effects and level of interaction severity are provided. A consumer and healthcare professional version of the interaction description are provided for each alert.

#### Electronic Medicines Compendium

Website: https://www.medicines.org.uk/emc/

The electronic medicines compendium (EMC) is an online resource containing information about drugs licensed for use in the United Kingdom. All website content is reviewed and approved by UK or European government agencies. The website includes: searchable database of Summaries of Product Characteristics (SmPC) for health professionals; Patient Information Leaflets (PILs); Risk Minimisation Materials (RMMs); safety alerts; side effect reporting tool through Yellow Card.

Contraindications, special warnings, precautions for use, interactions, incompatibilities, side effects, adverse reactions and overdose precautions are listed in the SmPC and PILs. The side effect reporting tool allows patients and healthcare providers to report suspected problems to the UK government of incidents or side effects that may be associated with a drug or supplement. Alert cards are available for drugs that outline signs and symptoms of a serious reaction. Risk Minimisation material outline the risk factors for a drug and provide information for clinicians to promote the safe use of a drug.

#### Epocrates (Plus or pro)

Website: https://online.epocrates.com/

Epocrates is a web-based resource which includes: database of drug monographs; index of drug interactions; therapeutic tables; interaction checker. Epocrates is also offered as a mobile resource, though this was not evaluated for quality in the present study.

Multicheck Drug Interaction Checker is a searchable drug interaction checker tool that reports on potential drug interactions and their level of severity. The resource also includes a built-in feature to search for drugs that may have caused a particular side effect in a user. Monographs include a list of drug interactions, safety/monitoring information, contraindications, cautions and adverse reactions.

#### Herb-Drug Interactions, NCCIH Clinical Digest

Website: https://nccih.nih.gov/health/providers/digest/herb-drug

This digest contains an index of 8 herbs with evidence-based information obtained from clinical studies. Each summary provides a short description of the common uses for the herb and lists potential interactions with other substances.

Each summary includes a brief description of potential herb-drug interactions. When available, clinical trials and studies that investigate interactions with the herb are listed. An overview of safety warnings or potential side effects of the herb are provided.

#### Herbs at a Glance

Website: https://nccih.nih.gov/health/herbsataglance.htm

Herbs at a Glance is a website that presents an index of fact sheets for a variety of herbs. Fact sheets provide information including: general background and overview; safety information; potential side effects; evidence and information from scientific studies; links to other resources.

Fact sheets provide warnings and safety information about the herb. When applicable, drug interactions are listed.

#### HIV Drug Interactions

Website: https://www.hiv-druginteractions.org/checker

HIV Drug Interactions is a web-based resource developed by the University of Liverpool to provide healthcare professionals, patients and researchers with education on HIV drug interactions. The website features: printable resources such as interaction charts or treatment selectors; fact sheets; video lectures; HIV drug interaction checker.

The searchable drug interaction checker tool provides alerts for potential interactions between drugs or CAM products used to treat HIV. Alerts outline the level of severity, quality of evidence and a summary of the interaction. PDF printouts of interaction tables are available. Drug fact sheets often include cautions regarding prescribing and dosage.

#### IBM Micromedex (includes DrugDex, Drug-Reax, AltMedDex)

Website: https://www.micromedexsolutions.com/home/dispatch/ssl/true

IBM Micromedex is an online resource for health professionals that includes; drug interaction checker; IV compatibility; drug comparison tool; dosing tools and calculators; searchable drug, disease and toxicology database of monographs; Carenotes (printable reports for patient education). Micromedex is also offered as a mobile resource called *mobile*Micromedex, though this was not evaluated for quality in the present study.

The searchable drug interaction checker tool provides alerts for potential interactions between drugs, herbs/supplements, food, alcohol, tobacco, pregnancy/lactation and allergies. Interaction reports include a short summary of the warning, onset, level of severity, available documentation, clinical management guide and the probable mechanism. Drug profiles outline toxicology, adverse effects, warnings and potential interactions.

#### Lexi-Natural (includes LexiComp)

Website: https://webstore.lexi.com/Store/Individual-Databases/Lexi-Natural-Products

Lexicomp is an online drug information database and resource for healthcare professionals and patients. The resource includes: drug monographs; clinical calculators; patient handouts; pill identification. Briggs Drugs in Pregnancy and Lactation contains drug monographs with information specifically for pregnant and lactating users. Lexi-natural is an extension of Lexicomp which provides information on over 415 natural products. Lexicomp is also offered as a mobile resource, though this was not evaluated for quality in the present study.

Professional monographs provide information on contraindications, interactions, toxicology, adverse reactions and pregnancy and fetal risk summaries. Patient education handouts provide information on precautions, side effects and signs that indicate the patient should contact their healthcare provider. Briggs monographs list evidence for risks to fetus and breastfeeding.

#### MedicinesComplete (includes Herbal Medicines; formerly known as the British National Formulary)

Website: https://about.medicinescomplete.com/publication/herbal-medicines/

Developed by the Royal Pharmaceutical Society, MedicinesComplete is an online resource of the British National Formulary (BNF). The resource includes: database of drug monographs; dosing information; drug interaction checker; professional development content; research and case studies.

The resource provides searchable information on drug interactions, adverse effects, safety warnings and contraindications. Stockey's Drug Interactions module provides reports on drug-drug, drug-herb and drug-food interactions. The Martindales adverse drug reaction checker provides severity ratings and support for healthcare providers to manage patients who present with adverse drug reactions.

#### Medscape

Website: https://www.medscape.com/

Medscape is a web-based clinical resource tool for healthcare professionals. The resource includes: medical news articles; educational tools (clinical briefs, patient cases, quizzes, e-learning courses, videos and other educational activities for CME credits); patient handouts; drug interaction checker; pill identifier; clinical calculators; health directories; interactive diagnostic modules; clinical guidelines; journal articles through MedLine; drug database. The database contains monographs on prescription drugs, over-the-counter products and herbal supplements. Medscape is also offered as a mobile resource, though this was not evaluated for quality in the present study.

Drug monographs list adverse effects, warnings and contraindications. The searchable drug interaction checker provides alerts for potential drug and/or CAM interactions. Interaction alerts outline the level of severity and whether an alternative therapy should be used.

#### Merck Manual

Website: https://www.merckmanuals.com/en-ca/

Merck Manual offers an online version of the hardcopy Merck Manual with 2 separate editions for healthcare professionals and consumers. Both editions of the web-based resource include: searchable database of drug monographs; news; information profiles for diseases and health topics; quizzes, podcasts and other resources. The Health Professional edition also includes: case studies and training materials; procedural videos; clinical calculators and other clinical resources. In addition, the consumer edition includes: index of symptoms; index of information for different medical emergencies.

Health Professional drug monographs list warnings/precautions, contraindications, adverse reactions, drug interactions, safety issues, allergy and idiosyncratic reactions. Patient education monographs list possible side effects and possible drug interactions.

#### National Cancer Institute

Website: https://www.cancer.gov/about-cancer/treatment/cam

This website uses NCI's Physician Data Query (PDQ) database to provide information on CAM drugs that are used to treat cancer patients. Each drug contains a separate summary for patients and health professionals. Both patient and healthcare professional drug summaries provide general information about the treatment as well as clinical trials and evidence from the scientific literature.

When sufficient evidence is available, drug summaries list potential interactions with other cancer drugs. Adverse effects are included in both the patient and healthcare professional summaries.

#### Natural Medicines (formerly Natural Medicine Comprehensive Database (NMCD) and Natural Standard Database (NSD))

Website: https://naturalmedicines.therapeuticresearch.com/

Natural Medicines is a web-based resource that includes: interaction checker; effectiveness checker; nutrient depletion, pregnancy and lactation checker; adverse event checker. Natural Medicines includes several sub-databases containing information on food, herbs and supplements, herbal combinations, drug manufacturers, sports medicine, health and wellness topics and comparative effectiveness. Natural Medicines is also offered as a mobile resource, though this was not evaluated for quality in the present study.

The searchable drug interaction checker tool provides interaction alerts which include the severity, likelihood of occurrence and level of significance for the potential interaction. Both the professional monograph and patient handout include safety concerns and precautions, interactions with drugs, herbs, food or lab. The professional monographs also provide information on toxicology and adverse effects.

#### OncoRx Database

Website: https://www.onco-informatics.com/oncorx/

Onco Rx is a web-based and mobile database of interaction information for oncology drugs, chemotherapy regimens and CAM products. Onco Rx is also offered as a mobile resource called OncoRx MI, though this was not evaluated for quality in the present study.

The database provides information on the pharmacokinetic and pharmacodynamic properties of oncology specific drug-drug or CAM-drug interactions. Drug interaction reports are comprised of theoretical and evidence-based information.

#### PEPID Drug Information Database

Website: https://www.pepid.com/

PEPID Knowledgebase is a web-based database hosted through PEPID Connect. Features of PEPID Connect include: Drug interaction checker; drug-allergy checker; pill identification; IV Compatibility tool; lab manuals; clinical calculators; drug database; differential diagnosis and symptom checker; news and alerts; PEPID PGx pharmacogenomic tool. PEPID is also offered as a mobile resource, though this was not evaluated for quality in the present study.

The searchable drug interaction checker tool provides interaction monographs which outline the mechanism of interaction, effects, level of concern and recommended actions. Drug monographs outline contraindications, cautions, interactions and adverse drug reactions.

#### RxList (owned by WebMD)

Website: https://www.rxlist.com/script/main/hp.asp

RxList is a web-based resource that includes: health and drug news; symptom checker; drug interaction checker; articles on diseases and conditions; medical dictionary; media (quizzes, slideshows and images); pill identifier; drug databases. The website contains a database of monographs on vitamins, herbs and dietary supplements. Monographs contain a description of the drug, evidence for effectiveness, how the drug works, warnings, interactions and dosing recommendations.

Monographs list interactions, safety concerns, warnings and precautions. The searchable drug interaction checker tool provides alerts for potential interactions between 2 or more drugs or supplements. Interaction alerts are categorized according to level of seriousness and include separate sections for patients/caregivers and healthcare professionals.

#### RxMed

Website: https://www.rxmed.com

RxMed is a website for family physicians and patients that includes: database of disease monographs for common illnesses; database of monographs on prescription medication, herbs, vitamins and dietary supplements. Herbal monographs contain general descriptions, composition, medicinal uses, connected diseases and safety information.

Safety information is provided in the herbal monographs which include adverse side effects, contraindications and precautions.

### DISCERN Instrument Ratings

DISCERN scores presenting the means across two assessors (VM, HT) for 19 online resources are listed in Table 3 of “Appendix 2”. Of the 23 eligible online resources, 4 online resources (Onco-RX, Clinical Pharmacology, Epocrates and MedicinesComplete) were not accessible as they required paid subscriptions and were not available through the author’s institutions. The authors contacted the respective online resources to request access for the purposes of this study. Representatives from the online resources either did not reply or advised that they could not provide trial access. For this reason, these online resources were not assessed and are not included in Table 3 of “Appendix 2”.

**Table 3:** DISCERN ratings

	DISCERN Question	About Herbs	American Botanical Council	Caremark Drug Inter-actions	Drug Infor-mation	Drug Product Database	Drugs.com	Electronic Medicines Compendium	Herbs at a Glance	Herb-Drug Inter-actions	HIV drug Inter-actions	Lexi-Natural
Section 1: Is the publication reliable?	1. Are the aims clear?	5	5	5	1	4.5	5	3	5	5	5	5
	2. Does it achieve its aims?	5	5	5	1	3	5	3.5	5	5	5	5
	3. Is it relevant?	4.5	5	4.5	2.5	3	5	5	4.5	3.5	4	5
	4. Is it clear what sources of information were used to compile the publication (other than the author or producer)?	5	5	1	2	3.5	4.5	1.5	4.5	5	2.5	5
	5. Is it clear when the information used or reported in the publication was produced?	5	5	2.5	2	5	5	5	5	5	2.5	5
	6. Is it balanced and unbiased?	5	5	2.5	3	4.5	5	4	5	5	3.5	5
	7. Does it provide details of additional sources of support and information?	4.5	5	1	1	4.5	5	2.5	5	5	2.5	5
	8. Does it refer to areas of uncertainty?	5	5	3.5	3.5	5	5	3.5	5	5	5	5
Section 2: How good is the quality of information on treatment choices?	9. Does it describe how each treatment works?	5	5	3	2.5	3	5	5	1.5	2.5	2	4
	10. Does it describe the benefits of each treatment?	5	4.5	2	2.5	3	4.5	5	4.5	5	1	5
	11. Does it describe the risks of each treatment?	5	4.5	5	2.5	3	5	5	4.5	5	4	5
	12. Does it describe what would happen if no treatment is used?	1	1	1	1	1.5	1	1	1	1	1	1
	13. Does it describe how the treatment choices affect overall quality of life?	1	2.5	2	3	2.5	3	2.5	1.5	1.5	1	2
	14. Is it clear that there may be more than one possible treatment choice?	1.5	3.5	1	1.5	2	4.5	4	4	2.5	3	2.5
	15. Does it provide support for shared decision-making?	3.5	2.5	3.5	4	2	4	3	4	2	1	4
Section 3: Overall Rating of the Publication	16. Based on the answers to all of the above questions, rate the overall quality of the publication as a source of information about treatment choices	4	4	2	3	3	4.5	3.5	4	3.5	3	4.5
Total DISCERN score (sum of Q1–15)		61.00	63.50	42.50	33.0	50.00	66.50	53.50	60.00	58.00	43.00	66.50

	DISCERN Question	Med-scape	Merck Manual	Micro-medex	National Cancer Institute	Natural Medicines	PEPID Drug Information Database	RxList	Rx-Med	Mean score	SD
Section 1: Is the publication reliable?	1. Are the aims clear?	5	4.5	5	5	5	4.5	4.5	5	4.58	0.99
	2. Does it achieve its aims?	5	5	5	5	5	5	5	4.5	4.58	1.03
	3. Is it relevant?	4.5	5	5	5	5	4	5	4	4.42	0.75
	4. Is it clear what sources of information were used to compile the publication (other than the author or producer)?	1	4	4	5	4	2.5	4	2.5	3.50	1.41
	5. Is it clear when the information used or reported in the publication was produced?	1	5	4	5	5	2.5	5	2	4.03	1.41
	6. Is it balanced and unbiased?	4.5	5	5	5	5	2.5	5	3	4.34	0.94
	7. Does it provide details of additional sources of support and information?	1	5	5	5	5	1.5	5	1.5	3.68	1.71
	8. Does it refer to areas of uncertainty?	5	4.5	4.5	5	5	4	5	3.5	4.58	0.63
Section 2: How good is the quality of information on treatment choices?	9. Does it describe how each treatment works?	5	4	5	5	5	5	5	5	4.08	1.24
	10. Does it describe the benefits of each treatment?	5	5	4.5	5	5	5	5	5	4.29	1.22
	11. Does it describe the risks of each treatment?	5	5	5	5	5	5	5	5	4.66	0.73
	12. Does it describe what would happen if no treatment is used?	1	4	2.5	1	1	1	1.5	1	1.29	0.75
	13. Does it describe how the treatment choices affect overall quality of life?	1	3.5	5	2	1	1	1	1	2.00	1.09
	14. Is it clear that there may be more than one possible treatment choice?	1.5	5	4.5	2.5	5	2	3	3	2.97	1.25
	15. Does it provide support for shared decision-making?	1	3	4.5	5	4	4	3	1.5	3.13	1.18
Section 3: Overall Rating of the Publication	16. Based on the answers to all of the above questions, rate the overall quality of the publication as a source of information about treatment choices	3	4.5	5	4.5	4.5	3	3.5	3	3.68	0.79
Total DISCERN score (sum of Q1–15)		46.50	67.50	68.50	65.50	65.00	49.50	62.00	47.50	56.13	10.25

Notably, none of the 19 online resources received a perfect score (5) on all 15 DISCERN questions. Evidently, most online resources received a poor rating in at least one question with 84.21% of resources receiving a score of 1 in at least 1 of the 15 DISCERN questions. Total scores for DISCERN ratings ranged from 33.0 – 68.50, with an average total score of 56.13 (*SD* = 10.25) out of a maximum score of 80. The 3 online resources receiving the highest total DISCERN score were Micromedex (68.50), Merck Manual (67.50) and Drugs.com (66.50). The 3 online resources that received the lowest total DISCERN scores were Drug Information (33.00), Caremark Drug Interactions (42.50) and HIV Drug Interactions (43.00).

For each DISCERN question, mean scores from all online resources were calculated. The 4 questions that received the highest mean scores included whether the risks were described for each treatment (*Mean* = 4.66*, SD* = 0.73), whether the aims were clear (*Mean* = 4.58, *SD* = 0.99), whether the source achieved those aims (*Mean* = 4.58, *SD* = 1.03), whether the website referred to areas of uncertainty (*Mean* = 4.58, *SD* = 0.63) and if the resource was relevant (*Mean* = 4.42*, SD* = 0.75). The questions that received the lowest mean scores referred to discussion about no treatment being used (*Mean* = 1.29, *SD* = 0.75), how treatment choices would affect quality of life (*Mean* = 2.00, *SD* = 1.09), and discussion of whether there is more than one possible treatment choice (*Mean* = 2.97, *SD* = 1.25).

Notably, some online resources including Drug Information and Drug Product Database, indexed many drugs that did not contain any content or did not have a monograph available. As a result, the DISCERN scores were considerably lower for these online resources, for both the overall mean score and ratings for individual questions. The absence of content for these drugs may be due to a variety of potential reasons; the product may have been discontinued or taken off the market, inadequate information is available, or the online resource is not up to date. However, it was often the case that when monographs were available, they contained comprehensive and high-quality content. Nevertheless, the ratings were significantly lower for these online resources because the information provided was not consistent among all treatments.

### Trends identified across resources assessed

After observing the results from the DISCERN assessments, the authors identified 3 main trends across the resources assessed:

#### No treatment choice

Question 12 in the DISCERN instrument assesses the degree to which a resource provides an explanation of what patients could expect if they did not undergo treatment. The majority of the online resources in this study scored poorly on this question, with 15 of the 19 (78.95%) online resources receiving a score of 1 out of 5 on this question. Additionally, none of the online resources received a perfect score of 5 on this question. Most resources discussed other aspects of CAM drugs but did not make any acknowledgement or provide discussion regarding the alternative option of no treatment. Our results suggest that it is not yet common practice for health resources to discuss this facet.

#### Treatment effect on quality of life

Question 13 in the DISCERN instrument assesses whether a resource describes how a treatment would affect the overall quality of life of the user. Of all online resources assessed, 12 out of 19 online resources (63.16%) received a score of 2 or lower on this question. Few resources provided information regarding how treatment choices may impact a patient’s day-to-day activities and relationships with family, friends, and careers. It does not appear to be common practice for websites and online resources to discuss these aspects for CAM treatments.

#### Safety and adverse effect information

Question 11 in the DISCERN instrument assesses whether a resource discloses the risks of each treatment. This question received a mean score of 4.66 (*SD* = 0.73) across all online resources, with 17 out of 19 (89.47%) online resources receiving a score of a 4 or higher on this question. These results were unsurprising, as our inclusion criteria required that resources provide information on interactions or adverse effects. Safety information reported by online resources include risks, warnings, precautions, potential side effects, or potential interactions. This information is often disclosed in a product monograph or as an alert from an interaction checker tool. Interaction checkers were provided by 12 out of 19 (63.16%) online resources and provide a tool for the user to search for potential interactions between drugs, CAM, food, alcohol and other substances. DISCERN does not assess the accuracy or comprehensiveness of the content, therefore it cannot be certain that the online resources include complete and extensive lists of all potential risks of CAM treatments. However, our results suggest that all online resources in this study provide information on the safety and adverse effects of CAM to some degree.

## Discussion

The aim of this study was to identify web-based online resources providing patient health information about adverse interactions or side-effects associated with CAM, and conduct a quality assessment using the DISCERN instrument. While some of these resources require paid subscriptions, the majority are freely accessible to the public and can be easily found via a Google search. With such easily accessible information, the consequence is the ease at which false, misleading or inaccurate information can be disseminated to the public. This elicits several concerns for the safety and well-being of consumers, especially amidst the tendency for patients to trust health information found on the Internet [38, 39], as well as perceive CAM as safe, thus feeling the need to take less precaution [40]. In addition, healthcare providers and researchers may use these online resources to gather information about CAM treatment choices and management plans for patients. To address these long-standing concerns and needs, this study provides healthcare professionals, researchers and patients with a list of resources, evaluated for quality, which could be used to compile comprehensive and evidence-based information about interactions or adverse effects associated with CAM. This was achieved by using the DISCERN instrument as a quality appraisal tool for various online resources containing CAM-specific information. Results from the assessment exhibit a considerable range in quality of information across online resources.

### Information on treatment choices: quality of life and risk of no treatment

There were 2 questions that most online resources scored poorly on for quality. The first was in regards to the quality of discussion about what a patient could expect if they delay or refrain from the treatment entirely. It may be argued that this question is outside of the scope and intent of CAM resources, which primarily aim to present the available information concerning the use of CAM drugs. However, it is important for healthcare providers and patients to be aware of the potential benefits and risks that a patient may experience if they make the decision to forgo CAM or other treatments for their condition. Providing further information on this subject would facilitate more informed and holistic management strategies for healthcare providers and consumers. Online resources also consistently scored poorly on the question associated with the discussion about how a treatment may affect a patient’s quality of life. Understanding these potential impacts may change a patient’s choice in treatment or disease management, therefore, it is important that this information be communicated to the patient. The inclusion of these components does not appear to be common practice for many online resources.

### Target audiences

While the identified online resources containing CAM-specific information are generally designed to be accessed and used by clinicians, the users of this information includes a wider range of individuals, inclusive of researchers, patients and the general public. As the needs of these end-users will vary, content in the online resources should be reflective of the intended users. Healthcare providers require additional information that is irrelevant or too complex for the general public. For example, information on pharmacology and dosing instructions would be imperative for a healthcare provider to incorporate into their clinical practice and treatment plans. On the other hand, this information may not be required or appropriate for the general public. Additionally, there is a tendency for health information on the Internet to be written at a level that is too complex for a large proportion of consumers to comprehend, thus increasing the risk of physical, emotional or financial harm associated with content that is misunderstood or misinterpreted by patients and their families [41, 42]. Resources such as Merck Manual, About Herbs, Lexicomp, National Cancer Institute and Electronic Medicine Compendium were found to provide separate reading materials for both healthcare professionals and patients. Online resources and websites are either separated entirely or contain 2 versions of monographs for each treatment, thus ensuring that CAM information is delivered to the intended audience at an appropriate reading level. A future study may evaluate the readability of online resources containing CAM-specific information to compare reading levels with target audiences.

### Strengths and limitations

A significant strength of this study includes the fact that the results from the systematic search strategy in Ng et al.'s (2020) scoping review were used to identify eligible online resources containing CAM-specific information for the present study. This methodological approach allowed us to extract a comprehensive and extensive list of online resources that met our research criteria. Additionally, all three authors participated in a pilot assessment of a subset of online resources prior to the independent assessment of all online resources by two authors, therefore increasing the validity of the ratings and associated analyses.

Given that we did not assess the accuracy of the information in the online resources, this may be noted as a limitation to the study. However, this form of validation is outside of the scope of the DISCERN instrument. Assessing the accuracy of information would require the authors to research and verify the evidence and claims found in each resource, which reflects a warranted future direction. An additional challenge involved the fact that the DISCERN instrument was designed to assess the quality of consumer health information about treatment choices, while the focus of this study was on the quality of patient information about adverse interactions or side effects associated with CAM. To our knowledge, no instrument exists that specifically assesses this type of information, thus we deemed the DISCERN instrument to be the most suitable given that adverse interactions and side effects make up a large component of the patient information surrounding treatment choices. Additionally, only studies published in the English language were includd in this study. Likewise, only online resources written in the English language were assessed, thus excluding other potentially relevant online resources that were written in a non-English language or referenced in a article published in a non-English language. Another limitation of the study is that 4 of the eligible online resources were not assessed with DISCERN. Despite our best efforts, we were unable to obtain free access to these online resources. After reaching out to the developers or companies of the online resources to request access, we either received no response to our request or were advised that a trial subscription could not be granted.

## Conclusion

Informed by the systematic search conducted in a previously published scoping review, this study provides a summary and quality assessment of existing web-based online resources that provide information on CAM and the associated adverse interactions or side effects. The present study provides a comprehensive list of web-based online resources containing CAM-specific information that are readily available on the internet. Given the prevalence of CAM use in the population and the abundance of online resources available on the web, this justified the need for a content quality assessment of these online resources. To the best of our knowledge, our study is the first to use the DISCERN evaluation instrument to provide an overview of the content quality of these online resources. After reviewing the results of the DISCERN assessments, we have outlined recurrent gaps in the quality of these web-based online resources. The summaries and quality assessments of each online resource, along with a list of recommended resources, can benefit healthcare providers, researchers, and patients in selecting suitable web-based resources to obtain high-quality, evidence-based CAM information.

## Data Availability

All relevant data are included in this manuscript.

## Appendix 1

See Fig. 1.

## Appendix 2

See Tables 1, 2 and 3.

## Abbreviation

CAM: Complementary and alternative medicine
